# Attention Restores Discrete Items to Visual Short-Term Memory

**DOI:** 10.1177/0956797612457782

**Published:** 2013-04

**Authors:** Alexandra M. Murray, Anna C. Nobre, Ian A. Clark, André M. Cravo, Mark G. Stokes

**Affiliations:** 1 Brain and Cognition Laboratory, Department of Experimental Psychology, University of Oxford; 2 Oxford Centre for Human Brain Activity, Department of Psychiatry, University of Oxford; 3 Department of Physiology and Biophysics, University of São Paulo

**Keywords:** short-term memory, attention

## Abstract

When a memory is forgotten, is it lost forever? Our study shows that selective attention can restore forgotten items to visual short-term memory (VSTM). In our two experiments, all stimuli presented in a memory array were designed to be equally task relevant during encoding. During the retention interval, however, participants were sometimes given a cue predicting which of the memory items would be probed at the end of the delay. This shift in task relevance improved recall for that item. We found that this type of cuing improved recall for items that otherwise would have been irretrievable, providing critical evidence that attention can restore forgotten information to VSTM. Psychophysical modeling of memory performance has confirmed that restoration of information in VSTM increases the probability that the cued item is available for recall but does not improve the representational quality of the memory. We further suggest that attention can restore discrete items to VSTM.

Visual short-term memory (VSTM) frees behavior from direct stimulus dependency. By constructing a stable internal representation of the world, people can act on visual information that is no longer present in the environment. Although critical for flexible, goal-directed behavior, VSTM can represent only a very small proportion of an individual’s total sensory input (Phillips, 1974). Consequently, attention must be focused selectively to ensure that only the most relevant information is encoded, and maintained, within this limited cognitive space.

Capacity limits in VSTM determine both the quantity and the quality of memory representations (Cowan, 2001; Luck & Vogel, 1997; Palmer, 1990; Wilken & Ma, 2004). When only a small number of items need to be retained, increasing the number of items in VSTM (e.g., from one to two items) reduces the precision of memory recall (Bays & Husain, 2008; Zhang & Luck, 2008). This trade-off implies that memory resources can be allocated flexibly among more than one item stored in VSTM to maximize mnemonic precision, given the available resources. Some researchers have argued that mnemonic precision can be subdivided ad infinitum to accommodate any increase in the number of representations in VSTM (Bays & Husain, 2008). Other researchers, however, have maintained that the number of items that can be stored in VSTM is limited (Zhang & Luck, 2008). Limiting the number of items encoded into VSTM could ensure that at least some memories are represented well enough to guide behavior successfully (Stokes & Nobre, 2012).

The key mnemonic parameters—quantity and quality—can be modeled using a task assessing memory precision in VSTM (Bays & Husain, 2008; Zhang & Luck, 2008). Observers are able to detect finer differences between a memory item in VSTM and a memory probe if the representational quality of the memory item is high than if the representation is less precise. Mnemonic precision, therefore, is quantified by the slope of the psychometric function relating accurate recall to the degree of difference between the memory item and the probe item (change magnitude). In contrast, if an item has not been encoded in VSTM at all (Zhang & Luck, 2008) or has been completely forgotten (Zhang & Luck, 2009), observers should be unable to detect even very large differences between the memory item and the probe. Therefore, the guessing rate indexes whether an item is represented in VSTM, irrespective of the representational quality.

Recent studies have shown that preparatory attention increases the discrete probability that task-relevant items will be stored in VSTM, but attention does not appear to influence the representational quality of encoded items (Murray, Nobre, & Stokes, 2011; Zhang & Luck, 2008). However, because people live in an ever-changing environment, it is important for VSTM to remain flexible and under the influence of top-down control even after initial encoding (Chun & Johnson, 2011; Stokes & Nobre, 2012). Indeed, studies have shown that flexible control over VSTM does not end at encoding; instead, representations continue to be shaped during VSTM maintenance (Griffin & Nobre, 2003; Landman, Spekreijse, & Lamme, 2003; Makovski, Sussman, & Jiang, 2008; Matsukura, Luck, & Vecera, 2007). In these studies, all stimuli were equally task relevant at initial encoding, but attention cues that were presented during maintenance retroactively predicted which item would be probed at the end of each trial. The studies showed that retroactive cues (retro-cues) presented during maintenance significantly increased recall of the cued items. It is important to note that the effects of retro-cuing are not dependent on iconic memory (Astle, Summerfield, Griffin, & Nobre, 2011; Sligte, Scholte, & Lamme, 2008); rather, attention seems to modulate VSTM throughout the retention interval (Kuo, Stokes, & Nobre, 2012).

Using a novel manipulation of cuing in a VSTM-precision task (Figs. 1a and 1b), we tested which of the two mnemonic parameters can be influenced by top-down signals during the maintenance period. To preview our results, psychophysical modeling demonstrated that retro-cues presented during maintenance increase the likelihood of recall but do not improve recall precision. This selective effect is consistent with the hypothesis that attention protects items from being forgotten (Zhang & Luck, 2009). However, we further demonstrate that attention can also increase recall of items that would otherwise be unavailable to VSTM retrieval mechanisms.

**Fig. 1.**
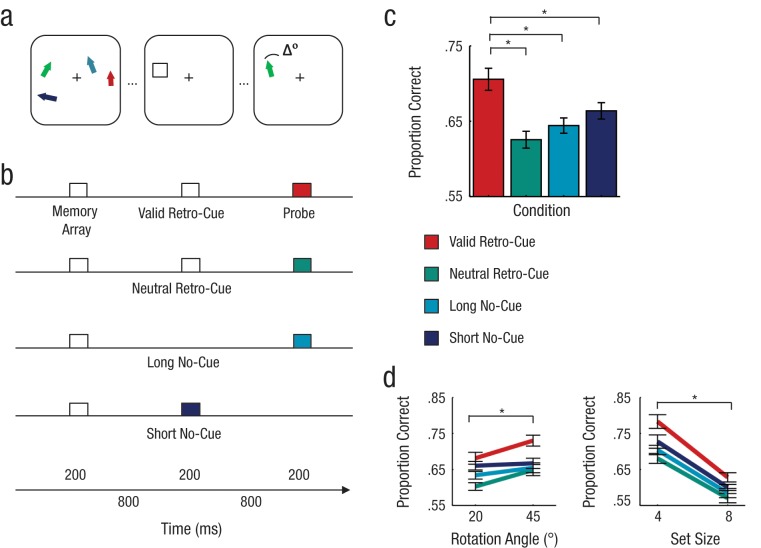
Study design (a, b) and main results (c, d) for Experiment 1. Participants were instructed to look at a monitor and remember the display, which consisted of an array of items such as those depicted at the left in (a). This display was presented for 200 ms. On half the trials, after an 800-ms delay, a square appeared, either in the center of the monitor (neutral retro-cue) or in the location where a memory probe would subsequently appear (valid retro-cue). The cue was presented for 200 ms and followed by an 800-ms delay. Finally, a single memory probe was shown, rotated either 20° or 45° clockwise or counterclockwise relative to the probed item. This probe appeared for 200 ms. On the remaining trials, there was no cue; instead, the memory probe was presented 800 ms after the offset of the memory array (i.e., at the same time that the cue appeared in the cued trials; short no-cue trials) or 1,800 ms after the offset of the memory array (i.e., at the same time that the probe appeared in the cued trials; long no-cue trials). The bar graph (c) shows the proportion of correct responses as a function of cuing condition. The line graphs (d) show the proportion of correct responses as a function of the probe’s angle of rotation (20° or 45°; collapsed across set size and across clockwise and counterclockwise rotations) and as a function of the set size of the memory array (4 items or 8 items; collapsed across angle of rotation). Asterisks indicate significant differences (*p* < .05). Error bars represent ± 1 *SEM*.

By using a memory probe to assess the contents of VSTM midway through the maintenance period, we found that, on average, fewer items were in VSTM at the time the retro-cue was presented than were subsequently available after retro-cuing. The retro-cue benefit, therefore, cannot be fully explained by an effective reduction in the demands on memory resources during the maintenance interval. Rather, our results show that retro-cuing increases the likelihood that items that would otherwise be lost or unavailable to VSTM are recalled. We suggest that attention may trigger an item to undergo a state transition from a non-VSTM format to a representation that is directly accessible for recall.

## Experiment 1

### Method

#### Participants

Twenty volunteers (ages 20–32 years) participated in Experiment 1. All participants had normal or corrected-to-normal vision, gave informed written consent, and were remunerated £8 (approximately US$13) for their time. The experiment was approved by the Oxford Central University Research Ethics Committee.

#### Task and stimuli

The task incorporated the retro-cuing paradigm developed by Griffin and Nobre (2003; see also Landman et al., 2003) with a VSTM-precision task (based on Bays & Husain, 2008) that we used previously (Murray et al., 2011). The task was programmed using Presentation software (Neurobehavioral Systems, Albany, CA) and was displayed on a 60-Hz CRT monitor at a distance of 100 cm. All stimuli were presented against a light-gray background, and a small black cross (0.86° × 0.86°) marked central fixation throughout each trial.

Each memory array consisted of a set of four or eight randomly oriented arrows in easily discriminated colors (red, blue, green, cyan, yellow, magenta, orange, or white; colors were randomly selected without replacement). The arrows subtended 1.43° of visual angle and were presented for 200 ms. Memory items were distributed evenly across the hemifields, and a minimum distance of 2.39° between items prevented overlap and reduced crowding effects.

Recall of one item was tested with a memory probe at the end of each trial. The memory probe was always one of the arrows from the memory array, rotated 20° or 45° around its central axis. By rotating the lever of a custom-built response device, participants indicated whether the probe had been rotated clockwise or counterclockwise. Accuracy feedback (i.e., whether the correct direction was selected) was presented for 300 ms.

On half the trials, a cue stimulus (an unfilled black square; 2.15° × 2.15°, 0.15° line thickness) was presented for 200 ms either at the location of the to-be-probed item (valid retro-cue; 25% of trials) or around the central cross (neutral retro-cue; 25% of trials). The cue was presented 800 ms after the offset of the memory array. After the cue disappeared, the fixation cross remained on-screen for a further 800 ms. The memory probe was then presented for 200 ms, and participants made their response.

The other half of the trials had no cue stimulus. On the short no-cue trials (25% of trials), the memory probe was presented after the first 800-ms delay; therefore, the memory probe in these trials appeared at the same time that the cues appeared in the trials with a valid retro-cue or neutral retro-cue. On the long no-cue trials (25% of trials), the memory probe was presented after 1,800 ms, a period equal to the two 800-ms delays plus the 200-ms cue period in the cued trials. These timing manipulations allowed us to estimate the contents of VSTM both at the time the cue was presented on retro-cue trials (short no-cue trials) and after the full delay as a function of cuing (valid retro-cue, neutral retro-cue, and long no-cue trials). After 20 practice trials, participants completed 768 experimental trials (trial type was randomized). Eye movements were monitored to ensure that participants maintained fixation.

Finally, in a follow-up experiment, we used the same stimuli as in the short no-cue condition, but varied the interval between the offset of the memory array and the onset of the memory probe (200, 400, 600, or 800 ms) to test whether significant forgetting of items occurred over the first 800-ms retention interval of Experiment 1. For more details, see the Supplemental Material available online.

### Results

Participants’ accuracy was analyzed using a 4 (cuing condition: valid retro-cue, neutral retro-cue, long no-cue, or short no-cue) × 2 (angle of rotation: 20° or 45°) × 2 (set size: 4 or 8) repeated measures analysis of variance (ANOVA). We found a main effect of cuing condition (Fig. 1c), *F*(3, 57) = 14.35, *p* < .001. Planned pairwise comparisons confirmed that accuracy was significantly higher in the condition with valid retro-cues, compared with all other conditions (all *p*s < .007). This result suggests that directing attention to items within VSTM enhances the mnemonic representation, thereby improving recall performance.

Performance in the short no-cue condition indexes the contents in VSTM at the time of the retro-cue, and evidence that accuracy was lower in this condition than in the condition with valid retro-cues demonstrates that the retro-cuing benefit cannot be explained by a simple reduction in the duration of the retention interval. Rather, we suggest that selective attention during maintenance can transform a relatively weak, or unstable, form of memory to a more robust representation to enable comparison with the memory probe (Makovski et al., 2008; Sligte et al., 2008). In this way, attention can be used to access information that would otherwise be unavailable or forgotten. Results from the follow-up experiment confirmed that there was significant forgetting between the offset of the memory array and the presentation of the cue (for more details, see the Supplemental Material). Retro-cues, therefore, could be useful for restoring information that has been forgotten.

As illustrated in Figure 1d, we also found main effects of set size (better performance for the set size of 4 than for the set size of 8), *F*(1, 19) = 201.35, *p* < .001, and angle of rotation (better performance for 45° rotation than for 20° rotation), *F*(1, 19) = 15.55, *p* = .001, as well as a marginal interaction between cuing condition and angle of rotation, *F*(3, 57) = 2.42, *p* = .076 (all other *p*s > .09). Retro-cuing was most effective when the judgment task was relatively easy (i.e., smaller set size, greater angle of rotation); this finding could indicate that attention improves the likelihood of recall of the cued item but does not increase the precision of the representation. This intriguing possibility motivated the design of the next experiment.

## Experiment 2

Twenty volunteers (ages 23–35 years) participated in Experiment 2. All participants had normal or corrected-to-normal vision, gave informed written consent, and were remunerated £8 (approximately US$13) for their time. The experiment was approved by the Oxford Central University Research Ethics Committee.

The purpose of Experiment 2 was to determine whether the cuing advantage observed in Experiment 1 was attributable to a change in the precision (or quality) of the VSTM representation or to a change in the probability that a memory would be recalled. We included a finer-grained parametric modulation of change magnitude to allow for formal modeling of parameters for precision and recall probability in each participant (Bays & Husain, 2008; Zhang & Luck, 2008).

### Method

#### Task and stimuli

The task was identical to the one used in Experiment 1, with two exceptions: To estimate the shape of the psychometric function relating recall to change magnitude, we tested a larger range of changes in angle of rotation: 5°, 15°, 30°, or 45°. To reduce trial numbers, we presented only a single set-size condition, with four items in each memory array. Thus, the experiment consisted of a total of 768 trials, presented across 10 blocks.

#### Modeling and analysis

**Fig. 2.**
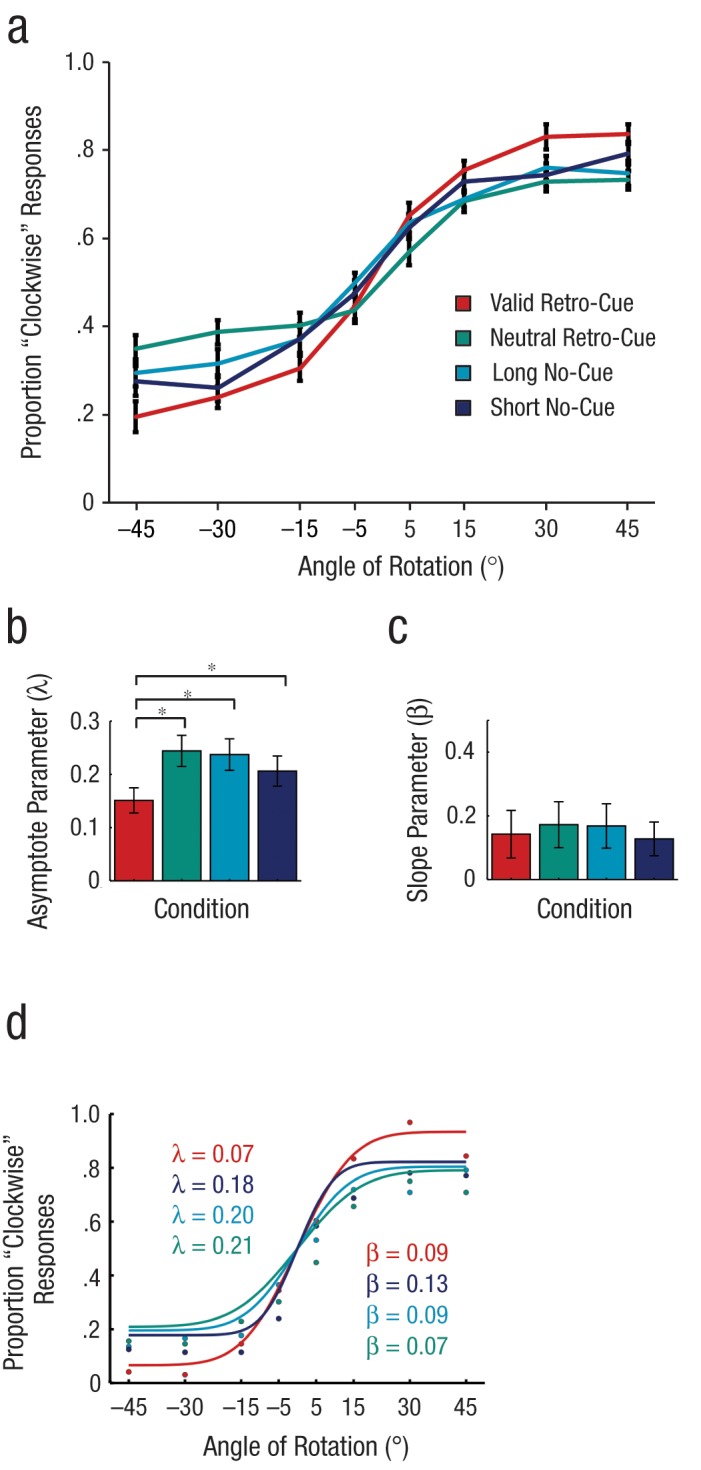
Behavioral results and parameter estimates for Experiment 2. In (a), the proportion of probes judged to have been rotated clockwise relative to the memory item is shown as a function of the probe’s angle of rotation for each of the four cuing conditions. The bar graphs show (b) estimates of the probability parameter (λ; lower numbers indicate higher probability of recall) and (c) estimates of the slope (β; higher numbers indicate greater precision) in each of the four cuing conditions. Asterisks indicate significant differences between conditions (*p* < .05). Error bars represent ±1 *SEM*. In (d), the proportion of “clockwise” responses in each condition is shown as a function of angle of rotation for 1 participant who completed four sessions of the task; the slope and asymptote parameters are also given for this subject. Each data point represents 96 trials (solid lines represent model fits).

The observed distributions of “clockwise” responses (i.e., responses indicating that the probe had been rotated clockwise relative to the memory item) were fit to a cumulative Gaussian curve for each participant and condition (Fig. 2a). Responses were modeled as binary outcomes, according to the following equation:

$$y=\lambda +\frac{\left(1-2\lambda \right)}{2}.erfc\left(\frac{-\beta }{\sqrt{2}}(x)\right),$$

where *erfc* is the complementary Gaussian error function, β is the slope of the curve, and λ is the asymptote of the curve (Murray et al., 2011). The asymptote provides an estimate of the probability that the probed item was represented in VSTM (Zhang & Luck, 2008), such that a lower value of λ indicates a higher probability that the item was in VSTM. Precision is indexed by the slope parameter (β): Steeper slopes reflect greater precision (Bays & Husain, 2008; Zhang & Luck, 2008).

### Results

As illustrated in Figure 2a, recall performance in all cue conditions increased as the angle of rotation increased; the curves reached plateaus when the angle of rotation reached ± 20° to 45°. Valid retro-cuing improved recall at these plateaus, which is consistent with a change in the asymptote of the psychometric function.

We found a significant main effect of cuing condition on the asymptote, *F*(3, 57) = 3.54, *p* = .02 (Fig. 2b), which we attributed to a smaller average asymptote parameter (λ) for the condition using a valid retro-cue, compared with all other conditions (all *p*s < .037). In contrast, no evidence suggested that attention influenced the precision (β) of representations (Fig. 2c; no main effect of cue type, *F* < 1). In the Supplemental Material, we compare models estimated for individual participants who completed three or four testing sessions (see Fig. 2d for results for 1 participant).

## Discussion

Our results demonstrate that attention, when directed to items already encoded in memory, improves the probability of their recall but does not increase the precision with which they are represented. Moreover, attention can rescue information that would otherwise be lost or unavailable to retrieval processes.

These results are at least partly consistent with the hypothesis that attention during VSTM maintenance protects behaviorally relevant information from interitem competition. According to this hypothesis, competitive dynamics similar to those that underlie perceptual processing continue to influence mnemonic delay activity in the visual system (Edin et al., 2009). Such winner-take-all network dynamics could help organize VSTM to represent coherent task-relevant objects (Duncan, 1998), but could also contribute to forgetting in VSTM (Edin et al., 2009). Memory items that lose competitive advantage would be crowded out by the presence of other items in VSTM; this suppression would result in a rapid return of delay activity to baseline for those items, such that little or no trace remains of the forgotten information. Zhang and Luck (2009) have shown that increasing the maintenance interval reduces the overall probability of recall but does not reduce precision—findings that are consistent with this competitive account. This reduction in recall probability is likened to “sudden death” (Zhang & Luck, 2009), in which the forgotten item has been actively suppressed by other retained memories. Top-down attention mechanisms also appear to bias competition in visual delay activity to favor the retention of cued items (e.g., Kuo et al., 2012)—another finding that is consistent with this competitive account.

**Fig. 3.**
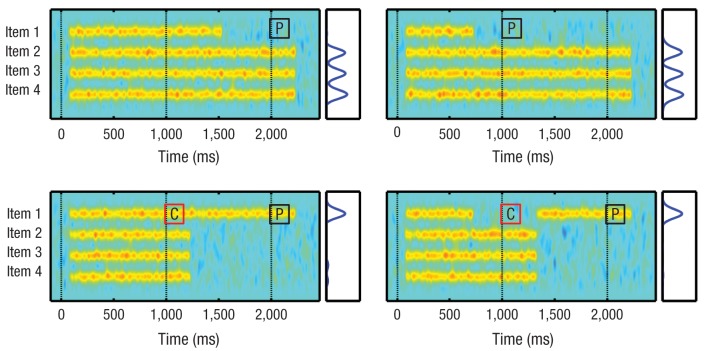
Illustrative depiction of our functional model showing persistent activation of neural representations corresponding to four memory items, as in our experiments (see also Edin et al., 2009). A *P* in a black square indicates the presentation of a memory probe; a *C* in a red square indicates the presentation of a cue. The top two panels represent a trial in which a memory item is forgotten late during the maintenance interval of a long no-cue trial (left) and a trial in which a memory item is forgotten early during the maintenance interval of a short no-cue trial (right). If a memory item is cued before it is forgotten (lower left panel), the delay activity may persist until the memory probe is presented, perhaps also allowing other task-irrelevant items to be forgotten and thereby reducing interitem competition. However, even if the item is already forgotten at the time of the cue, our results suggest that its representation in visual short-term memory can be restored by retro-cuing prior to the presentation of the memory probe (lower right panel).

In the present study, better recall performance in the valid retro-cue condition, compared with the two control conditions with the same retention interval (neutral retro-cue and long no-cue), could be explained by retro-cues creating a bias favoring representation of the cued items in VSTM, thereby increasing the likelihood that only the most task-relevant information will survive the hazards of forgetting. However, the evidence that recall probability was higher in the valid retro-cuing condition than in the short no-cue condition cannot be explained by top-down modulation of information already in VSTM. Results for the short no-cue condition provide an index of items in VSTM at the time of the retro-cue; thus, relative to the short no-cue condition (Makovski et al., 2008; Sligte et al., 2008), the valid retro-cue condition had an advantage in recall probability that demonstrated attention can increase access to information that would otherwise be unavailable to retrieval mechanisms. Moreover, psychophysical modeling has confirmed that this apparent “resurrection” of lost items operates discretely. Figure 3 presents four schematic examples of memory “delay activity” that illustrate what we suggest would occur during the various cuing conditions in our study.

We found no evidence that retro-cuing improves the representational quality of the cued item. Perhaps restoration to VSTM can be thought of as a complement to sudden death (Zhang & Luck, 2009), in that these processes reflect an item’s transformation from being available to being unavailable to retrieval mechanisms, and vice versa.

These results imply that some form of visual memory persists for seconds after an item has disappeared from view but that this memory may not be directly available for recall. Makovski et al. (2008) have proposed that attention protects items in VSTM from interference during retrieval and, in particular, from perceptual interference from the probe stimulus. This hypothesis suggests that attended items are raised to a special status within VSTM (see also Olivers, Peters, Houtkamp, & Roelfsema, 2011). Without attention, the information may not be stable enough to survive the retrieval process. Sligte et al. (2008) have proposed an intermediate buffer between iconic memory and VSTM. They suggested that many items remain represented in this intermediary high-capacity store, termed “fragile VSTM,” but that these representations are susceptible to interference and are not directly reportable. However, if attention is directed to items in fragile VSTM, those items can be transferred into the capacity-limited robust VSTM and become available for report (Sligte et al., 2008).

Our data provide further evidence that selective attention can increase the recall and reportability of specific items. Given the evidence that significant forgetting occurred prior to the presentation of the retro-cue, it appears that attention might be able to restore items that were previously reportable in VSTM but subsequently forgotten. However, it is also possible that attention can benefit items that were perceived but never fully encoded into a reportable VSTM format.

The effects of spatial attention during VSTM maintenance parallel the effects observed when attention is directed to items before they are encoded into VSTM. Specifically, preparatory attention increases recall probability but does not enhance memory precision (Murray et al., 2011; see also Zhang & Luck, 2008). Combined, these results suggest that attention does not affect the quality of representations in VSTM but does increase the probability that task-relevant items will be encoded and maintained.

In sum, our data show that selective attention during VSTM maintenance increases the probability that behaviorally relevant information will be recalled. Some of our results can be explained by a recall bias favoring items already in VSTM. However, our results also show that attention improves recall of items that would otherwise be irretrievable. By orienting attention to otherwise inaccessible representations, it may be possible to restore forgotten information to VSTM.
